# Determination of the Metabolites and Metabolic Pathways for Three β-Receptor Agonists in Rats Based on LC-MS/MS

**DOI:** 10.3390/ani12151885

**Published:** 2022-07-23

**Authors:** Ying Liang, Lin Wang, Ruipeng Zhang, Jiadi Pan, Wenhong Wu, Yuanyuan Huang, Zifan Zhang, Renbang Zhao

**Affiliations:** Faculty of Food Science and Technology, Agricultural University of Hebei, Baoding 071000, China; 15175898669@sina.cn (Y.L.); h15933935231@sina.com (L.W.); ruipeng_zhang@189.cn (R.Z.); panjiadi123@sina.com (J.P.); wwrx2152140@163.com (W.W.); 15933935231@163.com (Y.H.); zhangzifan2001@outlook.com (Z.Z.)

**Keywords:** salbutamol, clenbuterol, ractopamine, LC-MS/MS, metabolites, rats

## Abstract

**Simple Summary:**

Although β-receptor agonists are tightly controlled, there is still abuse. A general high-performance liquid chromatography-tandem mass spectrometry detection method was developed to measure three typical β-receptor agonists; the metabolites and metabolic pathways of SAL, CLB, and RAC in rats were investigated. The results showed that the metabolites in the rats mainly existed in the form of the prototype drugs and that they still had a few metabolites. The absorption and metabolism of the different β-receptor agonists in the rats were quite different, and the absorption and metabolism of the same β-receptor agonists in different tissues were also different. This will help to modulate the receptor agonist and to study its detection and metabolism in other species.

**Abstract:**

This paper developed a universal detection method by high-performance liquid chromatography-tandem mass spectrometry to detect three typical clenbuterols, CLB, SAL, and RAC, and to investigate the metabolism of β-agonists in vivo. The parent ions and daughter ions of the three β-receptor agonist standards and the residues in the muscle, liver, and blood samples of rats were obtained by Total Ions Scan mode. The metabolites produced in different tissues at a specific time were qualitatively and quantitatively analyzed, and the corresponding metabolic pathways were inferred. The results showed that the three β-receptor agonists mainly existed in the form of prototype drugs in rats, with a small amount of clenbuterol methyl compound and albuterol methyl compound. There were significant differences in residual metabolism between different tissues of the same species. In addition, different β-receptor agonists have different absorption and utilization rates in rats.

## 1. Introduction

β-receptor agonists are the generic name for a class of drugs, also known as “lean meat essences” in China because they can increase the lean meat rate of livestock and poultry. Among them, the three typical β-receptor agonists are Clenbuterol (CLB), Salbutamol (SAL), and Ractopamine (RAC) [1]. β-receptor agonists are generally used in the treatment of diseases such as asthma [2,3], but long-term use in poultry and livestock production has a significant “nutrient redistribution effect” that reduces fat accumulation and improves carcass leanness [4,5]. However, β-receptor agonists enter the human body through the food chain or active intake, and then, a series of adverse reactions, such as palpitations, tremors, and arrhythmias, occur, and excessive intake may even lead to sudden death [6,7]. It also has been shown that β-receptor agonists have specific side effects on the cardiovascular, liver and kidney, and reproductive systems and on skeletal muscle and the nervous system [8,9,10,11,12]. In the EU, legislation has been in place since the 1980s to regulate the use of growth promoters in animal production. In addition, China, Russia, and other countries have also banned its use [7]. However, the abuse of β-receptor agonists still exists in illegally adding feed, improving athletes’ performance, shaping bodybuilders, etc. [13,14].

The detection of β-receptor agonists plays an essential role in ensuring food safety. At present, detection methods, such as Enzyme-linked Immunosorbent Assay (ELISA), Capillary Electrophoresis (CE), Gas Chromatography-Mass Spectrometry (GC-MS) [14], and High-Performance Liquid Chromatography-Tandem Mass Spectrometry (LC-MS/MS), have been established to detect β-receptor agonists [15,16,17,18,19,20,21]. The Chinese national standard GB/T 22147–2008 and SN/T 4817–2017 standard detection method for CLB, SAL, and RAC is LC-MS/MS. The ELISA method is highly sensitive, simple, and convenient and can be quantitatively detected according to the depth of the color reaction [22,23]. It is often used as a rapid test and has developed rapidly in recent years. The CE method is simple and fast but inferior to the HPLC method in terms of injection accuracy and reproducibility [24]. The GC-MS method has good separation and determination results, but its development is limited by the complexity of the steps and the high cost of the derivatization reagents [25,26]. The LC-MS/MS method specified in the standard is relatively simple to operate, has high sensitivity, and is widely used for the qualitative and quantitative determination of veterinary drug residues [27,28].

**Figure 1:**
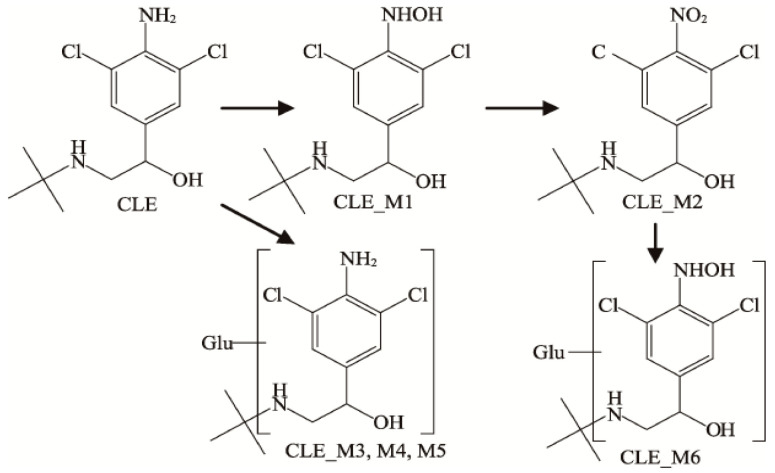
Metabolites in the urine of Clenbuterol.

β-receptor agonists produce different metabolites in different parts. Studies have shown that they are primarily in the form of glucuronic acid conjugates and oxidation products in animals [24,29]. The glucuronic acid binding of β-receptor agonists with the phenyl hydroxyl group mainly occurs in the phenyl hydroxyl group. Otherwise, it mainly occurs in the β-hydroxy group. Two kinds of SAL metabolites were detected in the plasma, namely the glucosylation and N-oxidation products of SAL, and five kinds of SAL metabolites were detected in the urine. In addition to the two products in plasma, there are benzene ring hydroxylation, benzene cyclomethoxylation, benzene ring hydroxylation dehydration [30], and small amounts of methyl compounds [31]. There are few studies on the metabolites of CLB in the edible parts of livestock and poultry, while studies in pig urine found six metabolites, which are the CLB prototype drug, product M1 (4-N-hydroxy CLB), and product M2 (4-NO_2_- CLB) and three isomers M3, M4, and M5 of the glucuronide binding product (Figure 1) [32]. RAC has been metabolized in the tissues, blood, and urine of sheep, rats, pigs, dogs, and turkeys, and it was found that most of those in the liver are in the form of the original drug, and the rest are RAC glucuronide conjugates (monoglucuronide and diglucuronide) [33,34]. However, a ractopamine mono-sulphate conjugate and a ractopamine mono-sulphate/mono-glucuronic acid diconjugate were detected in rat bile, which has not been detected in other species [35]. Some studies have found no residues in the tissues of ducks [36].

β-receptor agonists undergo complex metabolic and translational processes after being introduced into domesticated animal species. Most research has focused on establishing detection methods, but little research has been conducted on the biotransformation pathways and metabolites. The metabolic pathways and residues also differ across species and organs. After detection of SAL in broilers and guinea pigs, it was found that the residues in liver and kidney were generally higher than those in fat and muscle, and the residues can still be detected after a long time [37,38]. The residual concentration in urine was more significant than the residual concentration in plasma in beef cattle. SAL was absorbed rapidly in sheep, and the hair has a longer residual time than plasma and urine, and the content can still reach 5.70 ng/mL at 21 days [39,40]. During CLB administration, the residual metabolism in pig hair increased with the increase in the dosage, and the residues during the withdrawal period of 32 days were 87 μg/kg [41]. When CLB was administered orally, it was no longer detected in the muscle on day 14 of discontinuation [42]. CLB is rapidly absorbed in chicken, and the order of CLB concentration in chicken during the zero-dose period is: lung > liver > kidney > fat > muscle. Perdu-Durand [43] used 14C-labeled molecule and radio-HPLC quantitation to study the metabolites of CLB in liver microsomal fractions and precision-cut liver slices of rats and cattle and found that the 4-N-oxidation of CLB is the primary metabolic pathway for both species.

This article establishes a reliable, accurate, and simple high-performance liquid chromatography-tandem mass spectrometry. SAL, CLB, and RAC were administered to rats by gavage, and liver, kidney, muscle, plasma, and feces were collected at different time points after a single administration. The metabolites were analyzed and identified, and MS/MS secondary mass spectra inferred their cleavage pathways. At the same time, the metabolism of β-receptor agonists in the body was summarized, which will help to find out if the new harmful products are residual, which will benefit food safety.

## 2. Experimental

### 2.1. Reagents and Materials

Both the chromatographically pure methanol (CAS No. 67-56-1) and the acetonitrile (CAS No. 75-05-8) were purchased from Mindray (Beijing, China) Technology Co., Ltd. Hexane and formic acid (chromatographic grade) were obtained from Comio (Tianjin, China) Chemical Reagent Co., Ltd. Ammonia, chloral hydrate, and ethyl acetate were purchased from Damao (Tianjin, China) chemical reagent Ltd., Fuchen (Tianjin, China) chemical reagent Ltd., and Jinfeng (Tianjin, China) Chemical Co., Ltd. The analytical standards of Salbutamol (CAS No. 18559-94-9), Clenbuterol (CAS No. 21898-19-1), and Ractopamine (CAS No. 90274-24-1) were all purchased from Yuanye (Shanghai, China) Biotechnology Co., Ltd. Β-glucuronidase (Production Batch No. SLBW5844) was purchased from Sigma company. Pork and mutton were purchased at Baoding Market. A 3 cc/mL MCX extraction column was purchased from American Waters Corporation.

### 2.2. Animals and Sample Collection

The study was carried out on 30 male SD rats, 8 weeks old, weighing 200 ± 20 g, from Beijing Huafukang Biotechnology Co., Ltd.; they were kept in a controlled environment (temperature 22 ± 2 °C, relative air humidity 6–10%) with good ventilation and food and water available at will. The rats were randomly divided into the blank group, the tissue group (liver, kidney, thigh muscle), the blood sample group, and the stool group. Each experimental group contained 5 animals and was given 1 μg/mL of CLB, SAL, and RAC mixed standard stock solution of 1.2 mg/kg·bw with sterile gavage needles. The blank group was not given drugs. At 3 h, 6 h, 9 h, 12 h, and 24 h after a single dose, the animals were anesthetized with 3 mL/kg of 10% chloral hydrate intraperitoneally, and the rats were dissected after being rendered painless. A blood sample of 2.5–3 mL was taken from the artery and stored in an EDTA blood routine tube, and then, excess tissue and fat were removed, and tissue samples (liver, kidney, and thigh muscle) were collected. After the collection, the samples were accurately labeled, placed in sealed bags, and stored at −18 °C for future use. Fecal samples were collected from the metabolic cage at 6 h, 12 h, 24 h, 36 h, and 48 h after a single administration, and the samples were then accurately numbered, placed in sealed bags, and stored for future use. The research experiments conducted in this article with animals or humans were approved by the ethical committee and the responsible authorities of our research organization(s), following all guidelines, regulations, and legal and ethical standards required for humans or animals.

### 2.3. Analysis of Salbutamol, Ractopamine, and Clenbuterol

#### 2.3.1. Sample Extraction and Pretreatment

Two-gram muscle samples (rat, pork, and mutton) that had been naturally thawed and chopped with sterilized scissors were accurately weighed and put into a 50 mL centrifuge tube. After sequentially adding 6.00 mL of ammonium acetate solution and 45.00 μL of β-glucuronidase solution, the solution was vortexed and mixed. Afterward, it was bathed at 37 °C for 18 h and cooled to room temperature. Then, 6.00 mL of 5% ammonia water-ethyl acetate solution (configured when used) was added, mixed, and sonicated for 15 min and centrifuged for 15 min, and the ethyl acetate layer was transferred into another 50 mL centrifuge tube. The above operation was repeated twice, and the three extraction liquids were combined. The solution was mixed after 4.00 mL of 2% formic acid aqueous solution (configured with deionized water) was added; the ethyl acetate layer was dried with nitrogen at 45 °C, a proper amount of n-hexane was added; the mixture was vortex mixed and centrifuged for 10 min; the upper-fat layer was removed, and the remaining layer was reserved for later use [44].

Two milliliters of plasma was placed in a 50 mL centrifuge tube; 3.00 mL of 0.20 mol/L ammonium acetate solution and 50.00 μL of β-glucuronidase solution were added and then mixed evenly. After a water bath at 37 °C for 12 h, 10.00 mL of methanol was added and centrifuged for 10 min, and the supernatant was prepared.

The muscle extract was activated in a 3 cc/60 mg Waters Oasis HLB MCX extraction column, passed through the column, and eluted in sequence. The eluent was dried at 40 °C with nitrogen; the residue was dissolved with 1.00 mL of 0.1% formic acid aqueous solution (configured with purified water), transferred to a 10 mL centrifuge tube, and centrifuged at 4000 r/min for 10 min; the supernatant was then muscle test solution. The plasma test solution was obtained according to the same procedure.

One gram of mixed feces was weighed into a 50 mL centrifuge tube; 6.00 mL of acetonitrile was added and mixed; it was sonicated for 15 min and centrifuged for 10 min; then, the supernatant was poured into another centrifuge tube. The above operation was repeated twice. The supernatants obtained three times were combined, blown dry with nitrogen at 45 °C, and redissolved with acetonitrile, and the sample solution was passed through a 0.22 μm filter membrane and was ready for testing.

#### 2.3.2. High Performance Liquid Chromatography-Tandem Mass Spectrometry Analysis

This test was performed by a high-performance liquid chromatography-triple quadrupole mass spectrometer (purchased from Agilent, Santa Clara, CA, USA). The system has the ZORBAX Eclipse Plus C18 column (2.1 mm × 50 mm, 1.8 μm). Mobile phases A and B were 0.1% (*v*/*v*) formic acid aqueous solution and 0.1% (*v*/*v*) formic acid acetonitrile solution. The chromatography method kept the initial mobile phase composition (2% B) constant for 5 min, after which the linear gradient changed to 30% B and was kept for 10 min at 50% B. The flow rate was 0.20 mL/min, and 5.00 μL of the sample was injected each time. The liquid chromatography outlet was connected to an Agilent triple tandem quadrupole mass spectrometry detection system. The system was equipped with an electrospray ion source (ESI) and uses positive ion scanning. The specific operating parameters were as follows: capillary voltage: 115 v; drying gas temperature: 300 °C; nebulizer: 35 psi; flow rate: 11.00 L/min; acquisition mode: full scan (MS2 Scan), selective ion scan (MS2 SIM), product ion scan (Product Ion Scan), and multiple reaction monitoring (MRM).

### 2.4. Method Validation

The standard stock solutions of SAL, CLB, and RAC were accurately measured and diluted with methanol step by step from low concentration to high concentration. The standard curve was drawn according to the test results, and the detection limit (LOD) was calculated. The processed samples of pigs, sheep, and rats were accurately weighted, three levels and three parallel batches were set up, and the recovery and coefficient of variation were determined.

## 3. Results

### 3.1. Methodological Evaluation

The method’s detection limit (LOD) was 0.0050 μg/mL, based on the signal-to-noise ratio (S/N = 3). The average recovery rate and coefficient of variation of the three β-receptor agonists are shown in Table 1.

The average recoveries of the three receptor agonists in rats at different levels of the addition of three concentrations were above 85%, while the coefficients of variation were less than 10%. It can be seen that the method has high accuracy and good reproducibility. The method was applied to the negative samples of pork and mutton; the average recoveries of the pigs and sheep obtained from Table 1 were above 89%, and the coefficient of variation was less than 9%, indicating that the method is also suitable for the detection of the three receptor agonists in pigs and sheep.

### 3.2. Mass Spectrometric Analysis of Three β-Agonist Standard Products

The SAL, CLB, and RAC standards were repeatedly measured by liquid chromatography-triple quadrupole mass spectrometry. In the full scan (MS2 Scan) acquisition mode, the parent ion and retention time of the three β-receptor agonists were determined. The excimer ions [M + H]^+^ *m*/*z* of SAL, CLB, and RAC were 240.1000, 277.0000, and 302.1000, respectively; production scanning was then performed on them, and the primary fragment ions obtained are shown in Table 2.

**Figure 2:**
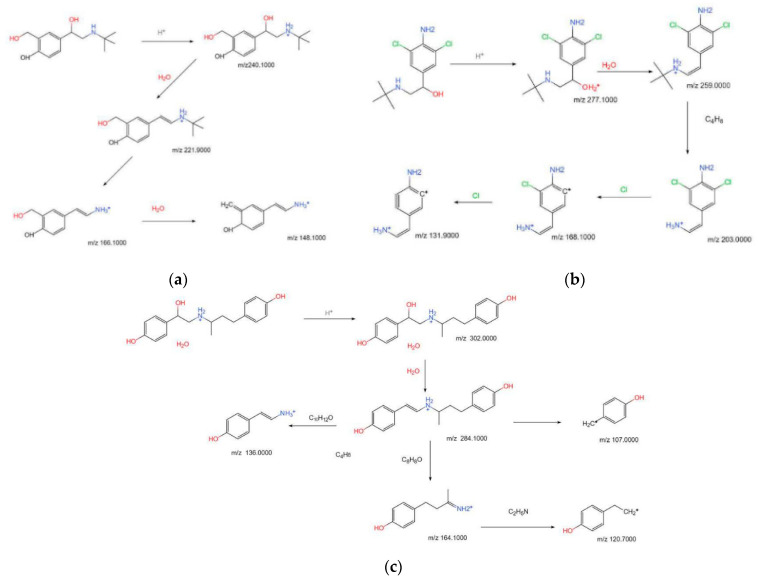
Pyrolysis pathway of SAL (**a**), CLB (**b**), and RAC (**c**) standards.

By comparing the mass difference of the fragment ions of SAL, it can be seen that *m*/*z* 240.1000 removes a molecule of water to form fragment ions *m*/*z* 221.9000 ([MH-H_2_O]^+^) and further removes 2-methylpropene (*m*/*z* 56) to obtain fragment ions *m*/*z* 166.1000 ([MH-H_2_O-C_4_H_8_]^+^), and the dehydration of *m*/*z* 166.1000 gives fragment ions *m*/*z* 148.1000 ([MH-H_2_O-C_4_H_8_-H_2_O]^+^). Because SAL has a hydroxy benzyl alcohol structure, a phenolic hydroxyl group, and an alcoholic hydroxyl group on its benzene ring, it is speculated that the dehydration reaction occurs between the phenolic hydroxyl group and the alcoholic hydroxyl group. The way of cracking is shown in Figure 2a.

The CLB’s first-level mass spectrometry shows that the abundance ratio of its excimer ions *m*/*z* 277.1000([H+M]^+^) and *m*/*z* 279.1000 are 3:2, the typical isotope peak containing two Cl atoms. The analysis of its second-level mass spectrometry showed that the Clenbuterol fragment ions were mainly *m*/*z* 259.0000, *m*/*z* 203.0000, *m*/*z* 168.1000, and *m*/*z* 131.900. Combining with the first-order mass spectrum, we can know that *m*/*z* 259.0000 ([MH-H_2_O]^+^) and *m*/*z* 203.0000 ([MH-H_2_O-C_4_H_8_]^+^) still contain *m*/*z* 261.0000 and *m*/*z* 205.0000, with an isotope abundance ratio of 3:2, which means that the chlorine atom is not lost in the cracking of this step. It can be inferred from the first and second mass spectrograms that the ions *m*/*z* 259.0000 ([MH-H_2_O]+) and *m*/*z* 203.0000 ([MH-H_2_O-C_4_H_8_]+) have similar cracking methods to Salbutamol; that is, it can be obtained, respectively, by removing one molecule of water from *m*/*z* 277.100 and further removing 2-methylpropene. While the fragment ions *m*/*z* 167.800 ([MH-H_2_O-C_4_H_8_-Cl]^+^) and *m*/*z* 131.900 ([MH-H_2_O-C_4_H_8_-Cl-Cl]^+^) have a mass difference of 35.1, which is 35.9 between *m*/*z* 131.900 and *m*/*z* 167.800, there is also no isotope peak of 3:2 in the first-level mass spectrum; so, it is speculated that the fragment ion *m*/*z* 167.800 ([MH-H_2_O-C_4_H_8_-Cl]^+^) and *m*/*z* 131.900 ([MH-H_2_O-C_4_H_8_-Cl-Cl]^+^) were, respectively, obtained by successively removing one chlorine substituent from fragment ion *m*/*z* 203.000 ([MH-H_2_O-C_4_H_8_]^+^). The way of cracking is shown in Figure 2b.

By comparing the mass difference between fragment ions *m*/*z* 302.1000 and *m*/*z* 284.1000 of Ractopamine, we can know that *m*/*z* 284.1000 ([MH-H_2_O]^+^) is obtained due to its parent ion *m*/*z* 302.0000 removing a molecule of water, the fragment ions are further cracked through three ways. The first was to remove C_8_H_8_O to obtain fragment ion *m*/*z* 164.1000 ([MH-H_2_O-C_8_H_8_O]^+^), and the second was to remove C_10_H_12_O to obtain fragment ion *m*/*z* 136.0000 ([MH-H_2_O-]^+^); the third was to obtain fragment ion *m*/*z* 107.1000 ([MH-H_2_O-C_11_H_15_NO]^+^) after cleavage; among them, the fragment ion *m*/*z* 164.1000 ([MH-H_2_O-C_8_H_8_O]^+^) continues to remove C_2_H_5_N to finally obtain the fragment ion *m*/*z* 120.7000 ([MH-H_2_O-C_8_H_8_O-C_2_H_5_N]^+^). The way of cracking is shown in Figure 2c.

### 3.3. Identification of Three Metabolites for β-Receptor Agonists

**Figure 3:**
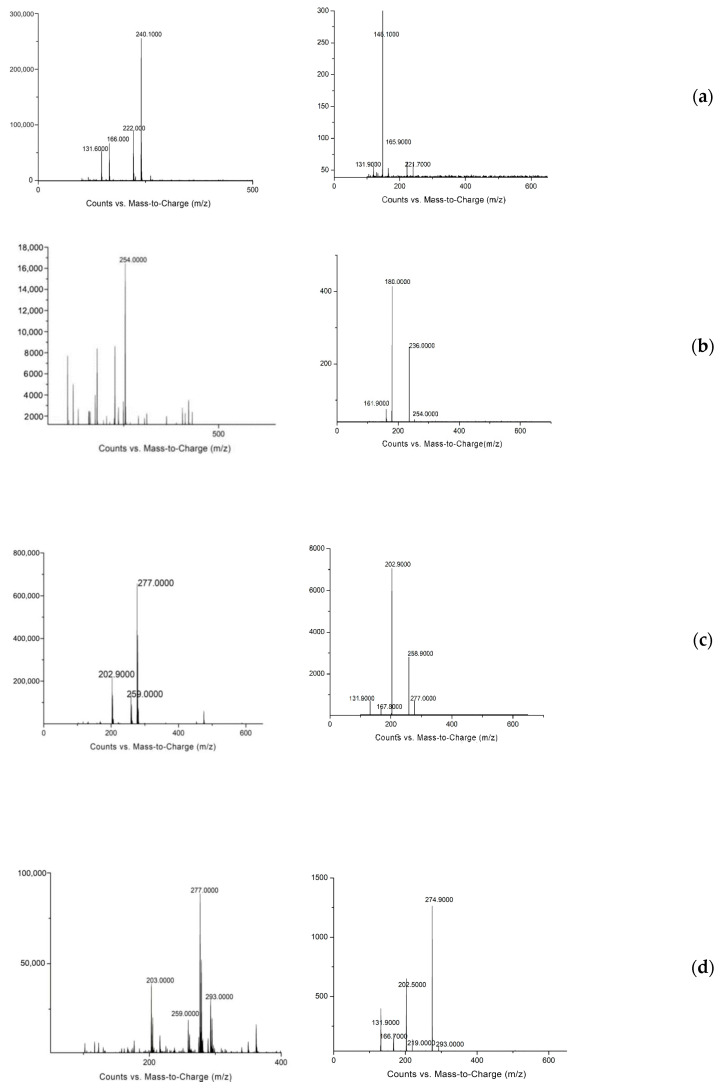

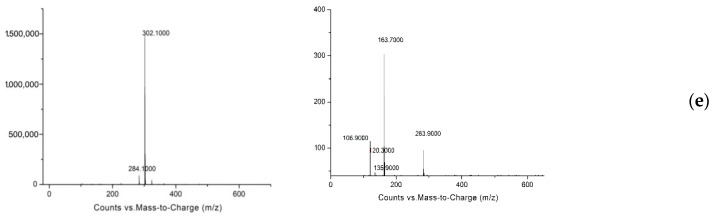
Primary mass spectrum (**left**) and secondary mass spectrum (**right**) of identified metabolites of three β-receptor agonists: (**a**) SAL-A, (**b**) SAL-B, (**c**) CLB-C, (**d**) CLB-D, (**e**) RAC-E.

In this experiment, rats were injected with SAL, CLB, and RAC at a dose of 1.2 mg/kg bw, and the plasma, liver, kidney, muscle, and feces of rats were collected at different time points. The samples to be tested were extracted and purified and then analyzed by mass spectrometry to determine the metabolites of the three β-agonist agonists, infer the cleavage pathway, and analyze the metabolic law. Comparing the first-order full scan images of the blank group and the experimental group, the prototype drugs of SAL, CLB, and RAC were found in the liver, kidney, muscle, blood sample, and feces. However, no metabolites of SAL and CLB were found (Figure 3). According to the reports in the literature, β-receptor agonists have complex metabolisms and low product contents in animals; so, selected ion scanning was further carried out. The relevant results are shown in Table 3.

In the selective ion mode, the methylated conjugates of SAL were found in the liver and kidney at 9 h and in the feces at 24 h, with a retention time of 5.043 min; CLB methylated conjugates were found in the kidney at 12 h and in the feces at 9 h, and the retention time was 5.971 min. According to the size relation of the response value, it was found that most of the residues in the rats were still in the form of the prototypical drugs of SAL, CLB, and RAC, while the methylated conjugates content of SAL and CLB was relatively low. Comparing the mass spectra shows that the original drug and its metabolites have very high similarities in chemical structure. Dewatering and removing 2-methylpropene are the same cleavage methods as for the SAL and SAL metabolites. Methpropene and successive removal of a chlorine substituent are the same cleavage methods as for the CLB and CLB metabolites.

### 3.4. Metabolism of Three Receptor Agonists In Vivo

**Figure 4:**
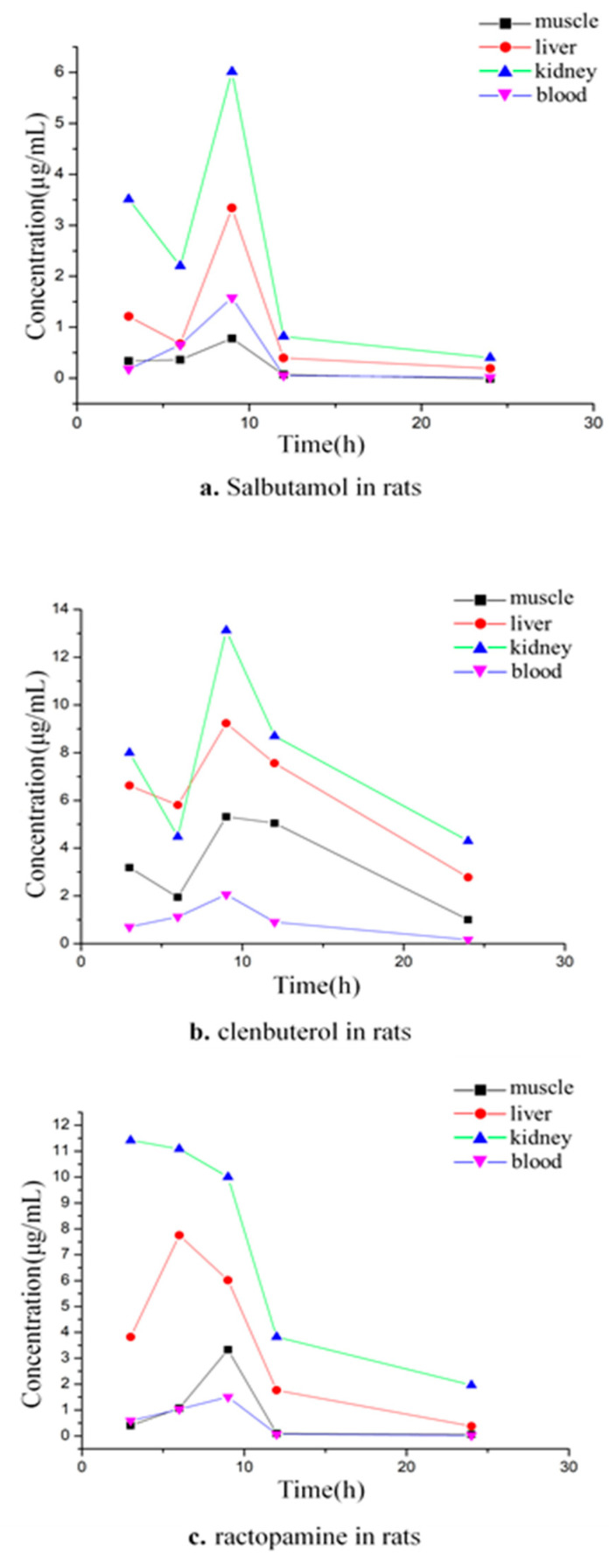
Relationship between concentration and time of SAL, CLB, and RAC in rat tissues.

There were significant differences in the absorption and metabolic residues of different agonists in different tissues of the same species. In the 3 to 24 h of the rats, SAL reached its peak concentration in the liver, kidney, muscle, and blood samples for 9 h (Figure 4a). According to the order of peak concentration from high to low, the tissues were arranged as follows: kidney, liver, plasma, and muscle. The peak concentration in the kidney at 9 h was 6.01 μg/mL, and the liver rose to reach a peak concentration of 3.34 μg/mL and then fell rapidly at 9 to 12 h. It can be seen that the kidney and liver absorb SAL quickly and digest it quickly. The rate of rising and falling in plasma is relatively gentle.

In the in vivo of the rats for 3 to 24 h, the time for the concentration of CLB in each tissue to reach the peak value was 9 h and from high to low was kidney > liver > muscle > plasma (Figure 4b). After the CLB enters the rat body by gavage, it can be quickly absorbed by the kidney and liver, and the concentration in 3 h can reach 8.00 μg/mL and 6.62 μg/mL; the concentrations of the peak value were 13.12 μg/mL and 9.23 μg/mL, respectively. The elimination rate of CLB in muscle was relatively slow at 9 to12 h after the peak, and it decreased rapidly at 12 to 24 h. The absorption and metabolism in the plasma were slow. 

During the 3 to 24 h in vivo of the rats, the RAC reached a peak concentration (11.42 μg/mL) in the kidney at 3 h, a peak concentration (7.76 μg/mL) in the liver at 6 h, and a peak concentration at 9 h in the muscle and plasma (Figure 4c). A longitudinal comparison of SAL, CLB, and RAC was carried out. According to the peak concentration, the various absorptions of the drugs and the utilization ratio were evaluated and ranked as CLB > RAC > SAL. By comparing the time to peak concentration, it can be seen that the absorption efficiency of RAC in the kidney is the highest. According to the comparison of the elimination trend chart, it can be seen that the fastest elimination rate of SAL and RAC was from 9 to 12 h, and from 12 to 24 h, it began to slow down. The elimination rate of CLB was faster during the 9 to 24 h, except for in the plasma. 

## 4. Discussion

β-Receptor agonists contain the parent structure of phenylethanolamine, from which it is easy to obtain H^+^ and form an [M+H]^+^ excimer ion peak in an ESI positive ionization mode, and it will lose neutral molecules such as H_2_O. Moreover, the (-C-N-) bond connected to the amino structure of these compounds will be broken, resulting in an ionic structure conjugated with the benzene ring (Ph-CH = CH-NH-). These two pairs of sub-ions have high abundance and are easy to detect; so, they can be used as “diagnostic” ions in the qualitative analysis of such compounds and their derivatives [45], which are the same as the test results. The structure of CLB contains Cl atoms directly connected with the benzene ring as aromatic chlorides, and the ơ band of the Cl atom will be cleaved in a neutral free radical manner during cleavage, which provides typical fragment ions [45].

Most of the residues in the rats were still in the form of the prototypical drugs of SAL, CLB, and RAC, while the methylated conjugates content of SAL and CLB was relatively low, which is consistent with the literature in that a small amount of the SAL methyl compounds was found in the plasma, and the original drug was detected [31]. However, researchers have found a small number of glucuronic acid conjugates of SAL, CLB, and RAC in the metabolite studies of cattle, goats, and rats [31,43,46], which was not detected in the experiment; the reason may be related to the metabolism between species. At the same time, due to the low single administration and low metabolite content in this study, it may have exceeded the sensitivity of the mass spectrometer and was not detected.

Perdu-Durand [47] detected radioactivity in metabolites by 14C labeling technology and HPLC technology, studied the metabolic pathway of HLB in rats and cattle, and found through the results of liver microsomal fractions and accurately cut liver slices that the 4-N-oxidation of HLB is the primary metabolic pathway of these two species, which is consistent with the results of this experiment.

Shi Jingfei [31] used high-performance liquid chromatography-mass spectrometry to detect the RAC prototype drug and the binding product of RAC glucuronic acid in pig and sheep urine. In this test, RAC prototype drugs were only detected in rats. Based on the above experimental data, it is preliminarily concluded that SAL, HLB, and RAC mainly exist as monobasic combined metabolites in rats.

The absorption and metabolism of different β-receptor agonists in rats are quite different, and the absorption and metabolism residues of the same β-receptor agonist in different tissues are also different [43,48,49]. The residues of SAL in broilers are liver > kidney > muscle [37]; the concentrations of SAL in pig liver and kidney 6 h after enzymolysis treatment are: 37.49 ± 4.61 μg/kg and 37.98 ± 4.61 μg/kg, respectively; the concentrations in the liver and kidney of sheep 6 h after enzymolysis treatment were 55.08 ± 14.8 μg/mL and 65.49 ± 6.75 μg/mL, respectively. Furthermore, the residual contents were higher than those in the plasma and muscle during the same period. After 24 h, the concentration of the residual amount in each tissue of cattle and sheep was liver > kidney > plasma > muscle; salbutamol accumulates quickly in the kidney and is eliminated quickly, and the residual elimination in the liver is slower than that in the kidney [31]. The above test results are consistent with the residual concentration at the initial stage of drug withdrawal and the clearance rate of the liver and kidney in this test, but the residual amount in the liver is different from that in the kidney 24 h after drug withdrawal [50,51,52]. The rules of residual metabolism are complex, and there is a certain relationship with the route of administration.

## 5. Conclusions

Although β-receptor agonists are strictly controlled, there is still abuse. Therefore, this study developed an LC-MS/MS method for detecting β-receptor agonists suitable for mice, pigs, and sheep. At the same time, the metabolites and metabolic pathways of SAL, CLB, and RAC in rats were studied, which will help the regulation of receptor agonists and their detection and metabolism studies in other species. Five metabolites were identified, of which three were drug prototypes, and two were methyl compounds of SAL and CLB. The metabolites in rats mainly exist in the form of prototype drugs, and they still had a few metabolites. The order of absorption and utilization of the three receptor agonists in rats was CLB > RAC > SAL. By combining this experiment and previous studies, it can be seen that the types of metabolites have a special relationship with the metabolism of the species, and the residual metabolism rules have a special relationship with the route of administration, which can be further studied.

## Figures and Tables

**Table 1:** Recovery rate and coefficient of variation of β-receptor agonist in rat, pig, and sheep.

Organization	Added Recovery Level (μg/mL)	n	Salbutamol		Clenbuterol		Ractopamine	
			Average Recovery (%)	Coefficient of Variation (%)	Average Recovery (%)	Coefficient of Variation (%)	Average Recovery (%)	Coefficient of Variation (%)
Muscle of rat	0.5	3	95.18	8.0	93.82	8.9	94.84	8.2
	1	3	94.22	8.9	92.52	4.8	90.55	7.1
	1.5	3	92.63	5.3	94.64	8.2	93.45	6.3
Liver of rat	0.5	3	91.71	4.1	92.63	6.3	89.85	5.2
	1	3	92.96	6.6	95.18	6.9	92.70	5.4
	1.5	3	89.85	7.9	94.23	8.9	88.95	5.2
Kidney of rat	0.5	3	92.05	6.5	94.70	7.9	91.63	7.0
	1	3	89.44	5.7	92.34	4.8	86.77	7.2
	1.5	3	91.18	7.8	90.87	7.7	90.45	7.4
Blood of rat	0.5	3	89.64	6.7	90.08	8.0	89.99	6.7
	1	3	88.21	6.2	90.15	7.0	88.47	6.1
	1.5	3	90.48	6.8	91.41	6.1	90.15	7.5
Feces of rat	0.5	3	92.54	9.3	89.00	7.3	89.69	7.0
	1	3	89.99	7.7	87.33	6.5	90.48	6.1
	1.5	3	87.00	6.9	88.66	7.4	87.78	7.4
Muscle of pig	0.5	3	95.26	6.3	96.45	6.0	89.96	7.8
	1	3	89.26	4.4	94.86	8.2	95.11	6.02
	1.5	3	95.63	5.9	96.73	6.1	92.97	3.5
Muscle of sheep	0.5	3	90.78	9.0	91.70	4.0	92.33	2.6
	1	3	90.12	3.6	94.08	7.4	89.56	3.9
	1.5	3	93.63	4.3	94.67	7.7	94.33	6.5

**Table 2:** Standard LC-MS/MS parameters for Salbutamol, Clenbuterol, and Ractopamine.

Name of Lean Meat Essence	Chemical Formula	Measured Relative Molecular Mass [M + H]^+^	Retention Time (min)	Major Fragment Ions (*m*/*z*)
Salbutamol	C_13_H_21_NO_3_	240.100	4.069	221.9000
				166.1000
				148.1000
				131.8000
Clenbuterol	C_12_H_18_C_l2_N_2_O	277.100	6.734	259.0000
				203.0000
				168.0000
				131.9000
Ractopamine	C_18_H_23_NO_3_	302.000	6.264	284.1000
				164.1000
				136.0000
				120.7000
				107.0000

**Table 3:** Three possible metabolites of β-receptor agonists.

Name of β-Receptor Agonists	Product	Forms of Metabolites	Measured Relative Molecular Mass [M + H]^+^
Salbutamol	A	Original form	*m*/*z* 240.1000
	B	Methylated conjugate	*m*/*z* 254.0000
Clenbuterol	C	Original form	*m*/*z* 277.0000
	D	Methylated conjugate	*m*/*z* 293.0000
Ractopamine	E	Original form	*m*/*z* 302.1000

## Data Availability

All data, models, and code generated or used during the study appear in the submitted article.
